# Broadband saturable absorption in germanene for mode-locked Yb, Er, and Tm fiber lasers

**DOI:** 10.1515/nanoph-2022-0161

**Published:** 2022-05-17

**Authors:** Qingbo Wang, Jianlong Kang, Pan Wang, Jiangyong He, Yicong Liu, Zhi Wang, Han Zhang, Yan-ge Liu

**Affiliations:** Institute of Modern Optics, Nankai University, Tianjin Key Laboratory of Micro-scale Optical Information Science and Technology, Tianjin 300350, China; College of Physics and Optoelectronic Engineering, Shenzhen University, Shenzhen 518060, China

**Keywords:** broadband saturable absorber, fiber laser, germanene, mode-locked

## Abstract

Passively mode-locked lasers have been widely investigated as one of the effective methods to obtain ultrashort pulses. As an important part of passively mode-locked fiber lasers, the exploration of 2D material-based saturable absorber has become one of the hotspots in ultrafast photonics in recent years. Germanene, a novel 2D Dirac material, with ultrafast optical response and broadband optical absorption, is a promising alternative material for saturable absorber in mode-locked fiber lasers. In this paper, germanium nanosheets are prepared via liquid-phase exfoliated method, with the saturable absorption property systematically characterized in three major wavebands of the near-infrared region. The generation of ultrashort pulses based on germanene saturable absorber in fiber lasers is further realized, in a broad waveband (1000 nm) centered at 1061.1, 1559.3 and 1883.5 nm, respectively. In addition, noise-like pulses operation with central wavelength of 1558.6 nm is also obtained, and the formation of rogue waves is further demonstrated via statistical analysis. To the best of our knowledge, this is the first experimental verification of the broadband saturable absorption property of germanene-based devices, covering three major fiber laser wavelengths from 1.0 to 2.0 μm.

## 1 Introduction

Ultrafast fiber lasers capable of delivering ultrashort pulses (e.g., femtoseconds or picoseconds) have been proved as the most effective tool for various crucial fields, such as strong-field physics, nonlinear optics, precision metrology, ultrafine machining, and industrial processing. Among them, passively mode-locked fiber lasers (MLFLs) stand out in the past decades, with the advantages of compact structure, self-starting operation, high stability, and free maintenance [1–6]. Importantly, saturable absorber (SA) is a necessary part in passively MLFLs, which can be divided into artificial SA and real SA [4, 7, 8]. For example, as an artificial SA, ultra-long-period grating, which has the advantages of compact structure, low price and reliable performance, has been successfully used to realize MLFL by Guo et al. [9]. The change of refractive index caused by strong light leads to phase mismatch, so that the strong light cannot be coupled into the cladding in the ultra-long-period grating, realizing the high transmittance output of strong light. By contrast, the real SA tends to have relatively simpler structures and broadband optical absorption property. Meanwhile, the research work about real SA is in the frontier and interdisciplinary fields of many disciplines such as physics, chemistry and material science. Therefore, the exploration of real SA has been a hotspot in the research of MLFLs in recent years. Since Bao et al. used graphene as real SA to achieve ultrashort pulses at 1565 nm in 2009 [10], it has become the focus of scientists to explore novel suitable real SA based on 2D materials, which have ultrafast carrier dynamics, 2D planar structures and broadband response [11]. Although a group of 2D materials have been successfully used as broadband SAs for MLFLs, such as graphene, black phosphorus (BP), transition metal dichalcogenides (TMDs), topological insulators (TIs), and MXenes, there are still some deficiencies that limit their practical applications [11]. For example, the development of graphene in the field of ultrafast photonics is hindered by its small modulation depth (MD) (2% for monolayer), while the application band of TMDs is limited by large bandgap, and BP with strong nonlinear characteristics has poor stability [12–17]. Therefore, it is necessary to further explore the low-dimensional materials with better performance for ultrashort pulse shaping.

Very recently, germanene has been shown to be a graphene-like Dirac material with ultrafast optical response and broadband optical absorption property, revealing promising applications in optoelectronic and phonic applications. Since the germanene layer is a natural quantum well, and the semiconductor layer acts as a reservoir to provide a relevant charge flow to the germanene layer, the carriers in the layer have a much shorter relaxation time. In addition, germanene also has a much higher nonlinear absorption coefficient than graphene, and its environmental stability is much higher than that of black phosphorus [18]. Therefore, germanene has great potential as a fast-SA for ultrafast photonics. However, due to the fabrication difficulties, there were very few reports on the application of germanene prior to 2018, especially in the field of optoelectronics. Only Matthes et al. predicted the optical conductivity and optical behavior with different photon energies of germanene through first principles in 2014 [19]. It is gratifying that Zhuang et al. successfully prepared germanene with the complete Dirac properties in 2018 [20]. Subsequently, Mu et al. in 2021 verified the saturable absorption property of germanene preliminarily and achieved ultrashort pulses centered at the wavelength of 1.5 μm [18]. As a Dirac material, germanene has the intrinsic property of broadband absorption. However, broadband (e.g., >500 nm) saturable absorption of germanene has not been demonstrated, as far as we know. Therefore, it would be interesting to experimentally explore its saturable absorption property and extend the application of germanene-SA to the broadband wavelength range of 1.0–2.0 μm.

Herein, germanium nanosheets are prepared by liquid-phase exfoliated (LPE) method, and then the laser-induced deposition method is used to deposit germanium nanosheets on the facet of an optical fiber connector to form the germanene-SA. Next, we experimentally investigate the optical property of the SA and verify its saturable absorption operation in all major fiber lasers (i.e., Yb-, Er-, and Tm-doped fiber lasers) in the wavelength range of 1.0–2.0 μm. Stable ultrashort pulses are realized at 1061.1, 1559.3 and 1883.5 nm, respectively. To the best of our knowledge, this is the first demonstration of the broadband (up to 1000 nm) operation property of germanene-SA, capable of generating ultrashort pulses covering three key wavelengths (i.e., 1.0, 1.5 and 2.0 μm).

## 2 Synthesis and characterization of germanium nanosheets

### 2.1 Fabrication of germanium nanosheets

The preparation process of 2D germanium nanosheet/*N*-methyl-2-pyrrolidone (NS/NMP) mixtures has been reported in our previous work [21]. The 2D germanium NS/NMP mixtures are centrifuged at a lower speed for a while to remove the germanium crystals of larger size, and then at a higher centrifugation speed for some time to obtain the desired 2D germanium nanosheets.

### 2.2 Characterization of the germanium nanosheets

**Figure 1:**
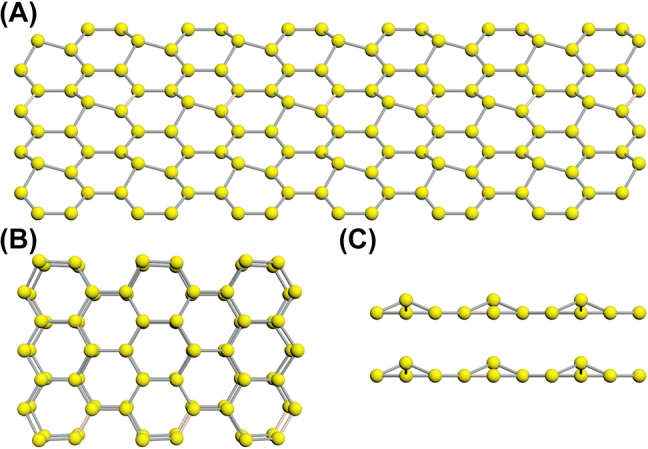
Structure of the germanium nanosheets. (A) The buckled honeycomb lattice of germanium nanosheet and its (B) top and (C) side views.

Germanium NSs are prepared by the classic LPE method. The structure of germanium NS is analyzed, and its structure diagram is shown in Figure 1. According to XRD data, the germanium crystals have a cubic diamond structure, which comprises of two interpenetrating face-centered cubic (FCC) sub-lattices, and each atom of these FCC sub-lattices is surrounded by four neighbours with equivalent covalent bonds between the atoms [22]. The germanene honeycomb lattice is buckled and composed of two vertically displaced sub-lattices, which is conforming to the Mermin–Wagner theorem [23].

Furthermore, the buckled structure should affect the intrinsic carrier mobility of germanene strongly, since the structure leads to a reduction of electron–phonon coupling strength [24]. Compared to graphene with a completely planar honeycomb lattice, germanene is theoretically predicted to have a larger energy gap due to its greater spin-orbital coupling strength [25].

**Figure 2:**
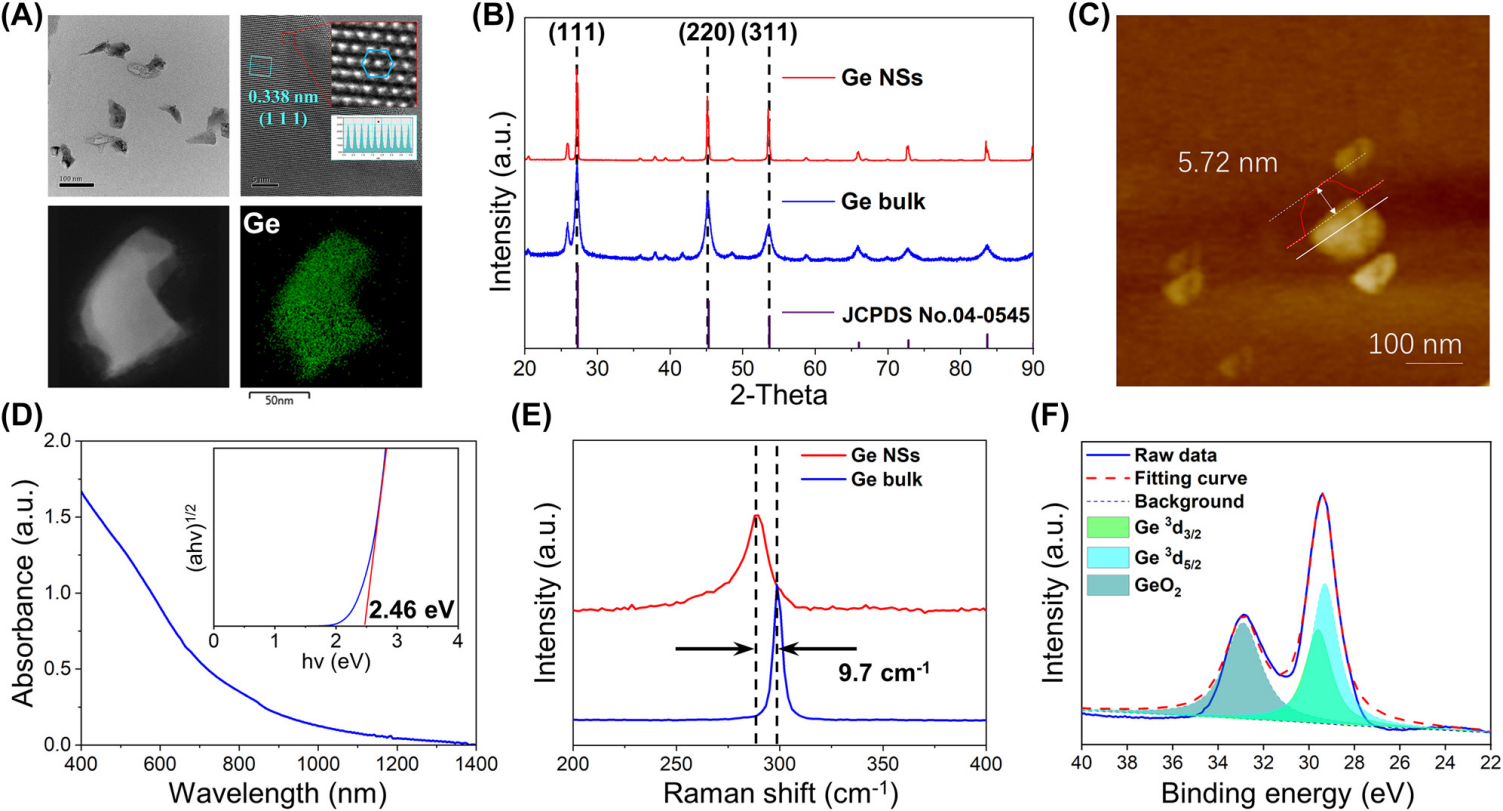
Characterization of the germanium nanosheets. (A) TEM image with shape, HR-TEM image with lattice and element mapping image of germanium NSs; (B) XRD spectrum of germanium NSs; (C) AFM image with thickness of germanium NSs; (D) UV–vis absorption spectrum of germanium NSs with optical bandgap; (E) Raman characteristic peak shift between germanium power and germanium NSs; (F) XPS spectrum of germanium NSs.

The microscopic morphology of the germanium NSs is characterized by transmission electron microscopy (TEM, FEI Tecnai G2 F30), which exhibits germanium NSs with an average lateral dimension of about 90.29 nm, as illustrated in Figure 2A. The good crystallinity of germanium NS in Figure 2B is further confirmed by X-ray diffraction (XRD, BRUKER D8 ADVANCE XRD equipment). All characteristic peaks of the fabricated germanium NSs (red curve) and germanium bulk (blue curve) are extremely congruent with the standard card of germanium (JCPDS No. 04-0545) which has *Fd*3*m* space group symmetry and lattice constants of *a* = *b* = *c* = 0.566 nm. As shown in Figure 2C, the thickness of the sample with the average 90.29 nm lateral dimension is 5.72 nm, as evaluated by atomic force microscopy (AFM, Bruker, Dimension ICON), corresponding to 15–20 layers of germanene [26, 27]. Optical absorption of the 2D germanium NSs depicted in Figure 2D is measured by a UV–vis–NIR spectrometer (Cary 60, Agilent), in which considerable broadband absorption range from 400 to 1400 nm can be observed. The optical bandgap of the germanium NS has been calculated to be 2.46 eV. The Raman spectral analyses of the germanium NSs and germanium bulk are employed by a high-resolution confocal Raman microscope (WITec alpha 300R) at 532 nm. Following the in-plane vibrations mode, the Raman spectra of germanium NSs reveal prominent peaks at 289.7 cm^−1^ (*E*_2g_). The in-plane vibrations mode (*E*_2g_) of germanium NS is redshifted by 9.7 cm^−1^ when compared to the bulk germanium powder, demonstrating the successful exfoliation of germanium NS and the structure of germanium NS (Figure 2E). The elemental composition and chemical state of germanium NS are explored by X-ray photoelectron spectroscopy (XPS, Thermo Fisher ESCALAB 250 Xi) with a monochromatic Al K X-ray source (1486.6 eV), as displayed in Figure 2F. The binding energies of the ^3^ *d*_5/2_ and ^3^ *d*_3/2_ doublets are located at 29.3 and 29.6 eV, respectively. The 32.9 eV sub-band is attributable to oxidized germanium (GeO_2_) which is oxidized during the preparation procedure. Some vacancies, flaws, and oxidation are unavoidable during the LPE of 2D materials [28]. The results are in accordance with what has been previously reported for germanium crystals [29].

## 3 Fabrication and characterization of germanene-SA

**Figure 3:**
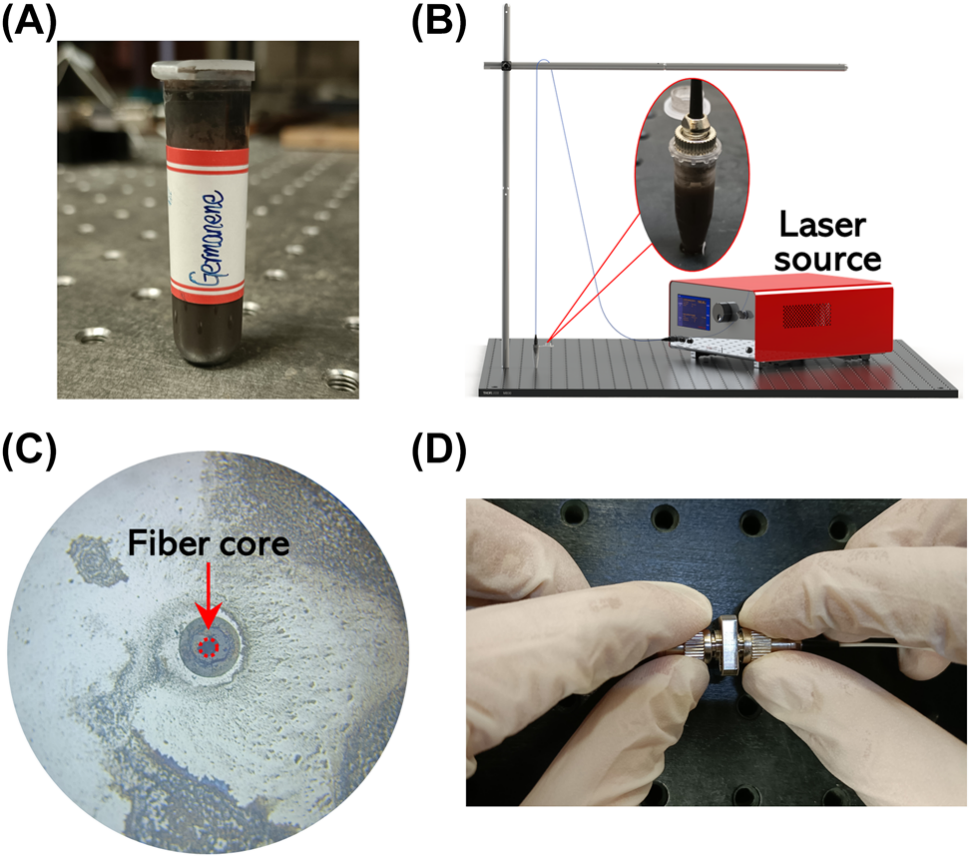
Optical deposition of germanium NSs. (A) Photograph of high concentration germanene dispersion; (B) experimental device diagram of laser-induced deposition method; (C) fiber facet with germanene after optical deposition; and (D) self-assembly conducted germanene-SA.

Germanium NSs are deposited on a single-mode fiber connector by the laser-induced deposition method [30, 31], and the experimental setup is shown in Figure 3B. The fiber connector is immersed into the germanene dispersion liquid which includes 1.5 ml isopropyl alcohol and 3.5 mg germanene, and the connector is further irradiated by a CW laser with a power of 70 mW for 25 min. Afterwards, the germanene is deposited on the fiber core area, enabling the interaction with the light propagating in the fiber. The micrograph of the fiber end face deposited with germanene is shown in Figure 3C. Finally, the germanene-SA is self-assembled by fixation of the two fiber connectors, as illustrated in Figure 3D.

**Figure 4:**
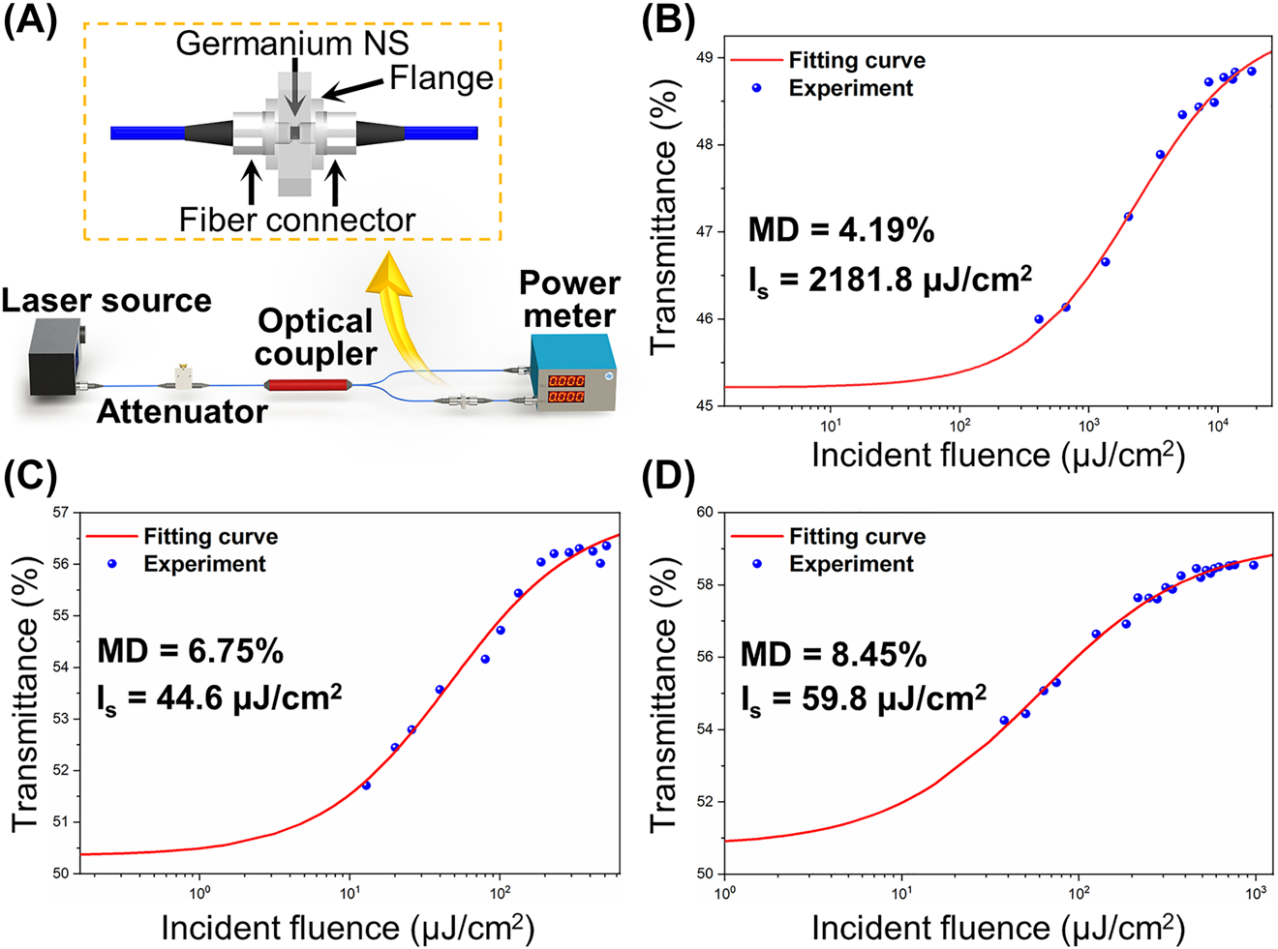
The measurement of nonlinear saturable absorption property. (A) The setup of balanced twin-detector measurement for the nonlinear saturable absorption property; (Inset: schematic diagram of the germanene-SA); the measured saturable absorption data and corresponding fitting curves under (B) 1031.6 nm; (C) 1564 nm; and (D) 1943.6 nm laser irradiation, respectively.

The nonlinear transmission characteristics of germanene-SA at the wavelengths of 1.0, 1.5 and 2.0 μm are studied by the balanced synchronous twin-detector measurement. The experimental setup is shown in Figure 4A. The laser source used at 1.5 μm is a commercial femtosecond fiber laser (Shanghai Langyan Optoelectronics Technology Co. Ltd., LF15608) with repetition rate of 49.5 MHz and pulse duration of 100 fs. In the 1.0 and 2.0 μm bands, home-made MLFLs (1031.6 nm, 610 ps, 4.6 MHz; 1943.6 nm, 807.5 fs, 46.15 MHz) are used. The maximum output power of laser sources used at 1.0, 1.5 and 2.0 μm are 15.5, 18.1 and 17.6 mW, respectively. Through continuous adjustment of the attenuator, the nonlinear transmittance of germanene-SA is clearly demonstrated, as a function of the incident fluence. The experimental data at the three wavebands could be fitted with the following formula:

$$T\left(I\right)=T_{\text{unsat}}+\Delta T-\frac{\Delta T}{1+I/I_{\text{sat}}}$$

where *T*(*I*) is the transmission, Δ*T* is the MD, *I* is the incident fluence, *I*_sat_ is the saturable fluence, and *T*_unsat_ is the unsaturable loss. Fitting results are shown in Figure 4B–D. At 1.0, 1.5 and 2.0 μm bands, the saturable incident fluence of germanene-SA are 2181.8 μJ/cm^2^, 44.6 μJ/cm^2^, and 59.8 μJ/cm^2^, respectively, while the MD are 4.19, 6.75, and 8.45%, respectively. In addition, the nonuniformity of germanium NSs deposited on the end face of the optical fiber will cause the optical scattering, resulting in the high nonsaturable loss of germanene-SA. These results indicate that the fabricated germanene-SA has the potential to be applied for mode locking in the 1.0, 1.5 and 2.0 μm regions.

## 4 Experimental setup

**Figure 5:**
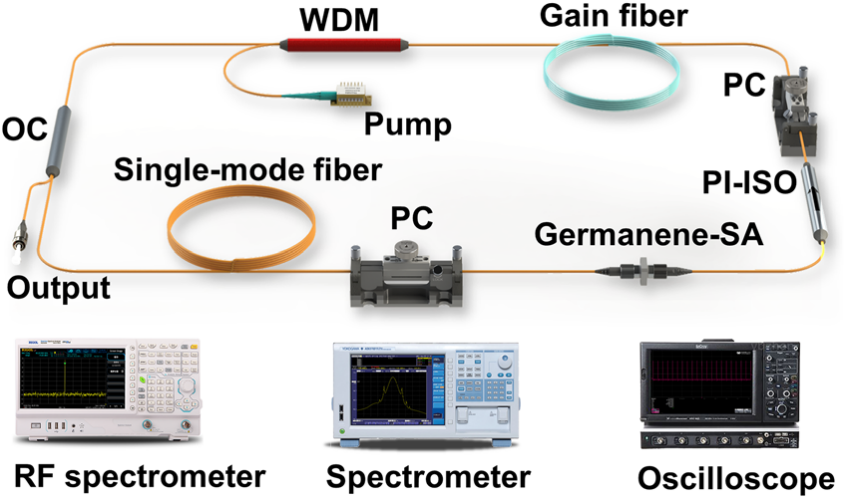
Experimental setup of germanene-SA based mode-locked EDFL and TDFL.

**Figure 6:**
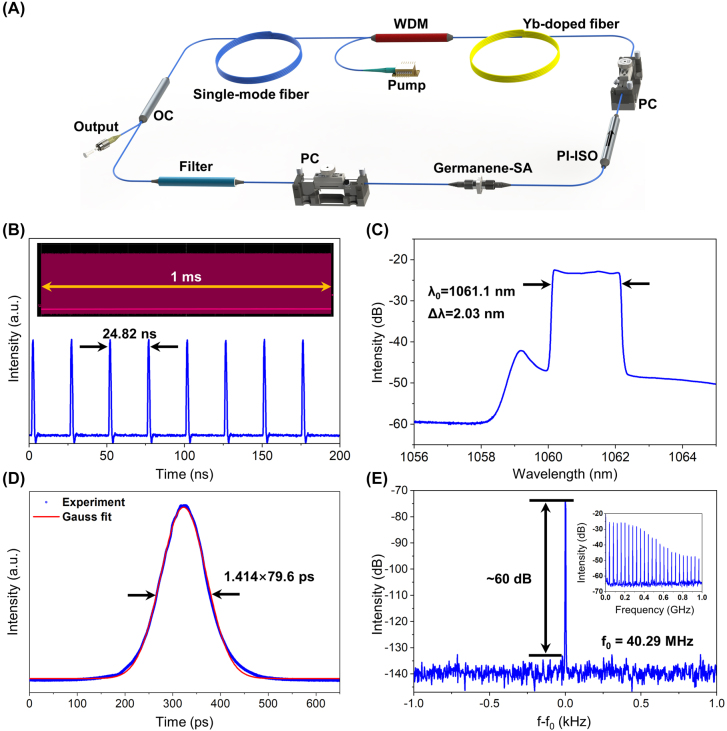
Dissipative soliton for mode-locked YDFL. (A) Schematic of germanene-SA based mode-locked YDFL; (B) oscilloscope trace; (inset: 1 ms pulse trains); (C) optical spectrum; (D) autocorrelation trace; (E) RF spectrum with 1 Hz resolution in the range of 2 kHz (inset: RF spectrum with 100 kHz resolution in the range of 1 GHz).

The beam path diagram, common to Er-doped fiber laser (EDFL) and Tm-doped fiber laser (TDFL), based on germanene-SA is shown in Figure 5. The pump light is emitted by the laser diode and injected into the gain fiber through a wavelength division multiplexer (WDM) (980/1550 nm WDM for EDFL, and 1550/2000 nm WDM for TDFL). In this experiment, backward pumping is used. The polarization insensitive isolator (PI-ISO) enables the unidirectional propagation in the cavity. Two polarization controllers (PCs) are employed to optimize the performance of the MLFL. Herein a piece of 0.5 m Er-doped fiber (LIEKKI, Er110-4/125) and a piece of 2.9 m Tm-doped fiber (Nufern, SM-TSF-9/125) are used as gain fibers of the EDFL and the TDFL, respectively. And 80:20 optical couplers (OCs) are used as the output couplers of both lasers. The rest of the cavities are composed of standard single-mode fibers (SMF28e). The experimental setup of Yb-doped fiber laser (YDFL) is shown in Figure 6A. A piece of Yb-doped fiber (LIEKKI, Yb1200-4/125) is backward pumped by a 980 nm pump via a 980/1064 nm WDM. A 90:10 OC is used for output, extracting 10% of the power for measurement. Since the MLFL at 1.0 μm band is in the all normal dispersion regime, a piece of 8 nm band pass filter (centered at 1064 nm) is inserted in the cavity for pulse shaping. The rest of the cavity is composed of standard single-mode fiber (HI1060).

In this work, a 45-GHz photodetector (DiscoverySemi, DSC10H) is used to detect the output signals of YDFL and EDFL, while a 12.5-GHz photodetector (Newport 818-BB) is used to detect the output signals of TDFL, which are further recorded by a 2-GHz oscilloscope (LeCroy, WaveRunner 620Zi). The YDFL and EDFL share the same optical spectrum analyzer (Yokogawa, AQ6370) to monitor the spectral information, meanwhile the output signal of the TDFL is monitored by another optical spectrum analyzer (Yokogawa, AQ6375). The RF spectra of the three bands are recorded by the same RF spectrum analyzer (Rigol, RSA3045), and the pulse duration is measured by an auto-correlator (Femtochrome, FR-103XL).

In the experiment, a high-speed oscilloscope (Tektronix DPO75902SX) is used to continuously collect the noise-like pulses to statistically analyze the generation of rogue waves.

## 5 Results and discussion

### 5.1 Mode-locked YDFL characterization

In the YDFL shown in Figure 6A, the total length of the cavity is about 5.13 m, including 0.6 m gain fiber, and the total group velocity dispersion (GVD) is calculated to be 0.13 ps^2^. Mode-locked fiber lasers with all normal GVD generally support dissipative soliton operation [32]. The germanene-SA based mode-locking is self-started when the pump power is increased above 280 mW. The results shown in Figure 6B–E are the output characteristics of the mode-locked YDFL when the pump power is at 320 mW. The time interval between pulses is 24.82 ns, which corresponds to the repetition rate of 40.29 MHz. The inset in Figure 6B is a long-term uniform pulse train over 1.0 ms, and there is no obvious fluctuation in the pulse intensity, indicating that the germanene-SA based mode-locking operation is in a state of high stability. The spectrum is centered at 1061.1 nm and the 3 dB bandwidth is 2.03 nm. In MLFL with normal GVD, stable pulses are realized by a comprehensive balance between self-phase modulation, dispersion, spectral filtering and saturable absorption, tending to produce M-type spectra similar to Figure 6C [33]. The autocorrelation trace of the mode-locked pulses is shown in Figure 6D, and the pulse duration is 79.6 ps, obtained by Gaussian fitting. Generally, the time bandwidth product (TBP) is widely used to estimate the chirp degree of the pulsed lasers [34–36]. The TBP of the YDFL pulse is calculated to be 43.19, much larger than the Fourier transform limit of 0.44 for a Gaussian pulse. A large TBP means that the dissipative soliton is strongly chirped, resulting in an enlarged pulse width. In Figure 6E, the repetition rate of the mode-locking pulses shown in the RF spectrum is 40.29 MHz, consistent with the total cavity length of the YDFL. And the signal to noise ratio (SNR) of RF spectrum is 60 dB at the fundamental repetition rate, indicating high stability. Finally, when the pump source reaches the maximum output power of 440 mW, YDFL can still be in a stable mode-locked state, and the average output power is 8 mW.

### 5.2 Mode-locked EDFL characterization

#### 5.2.1 Noise-like pulses in the mode-locked EDFL

**Figure 7:**
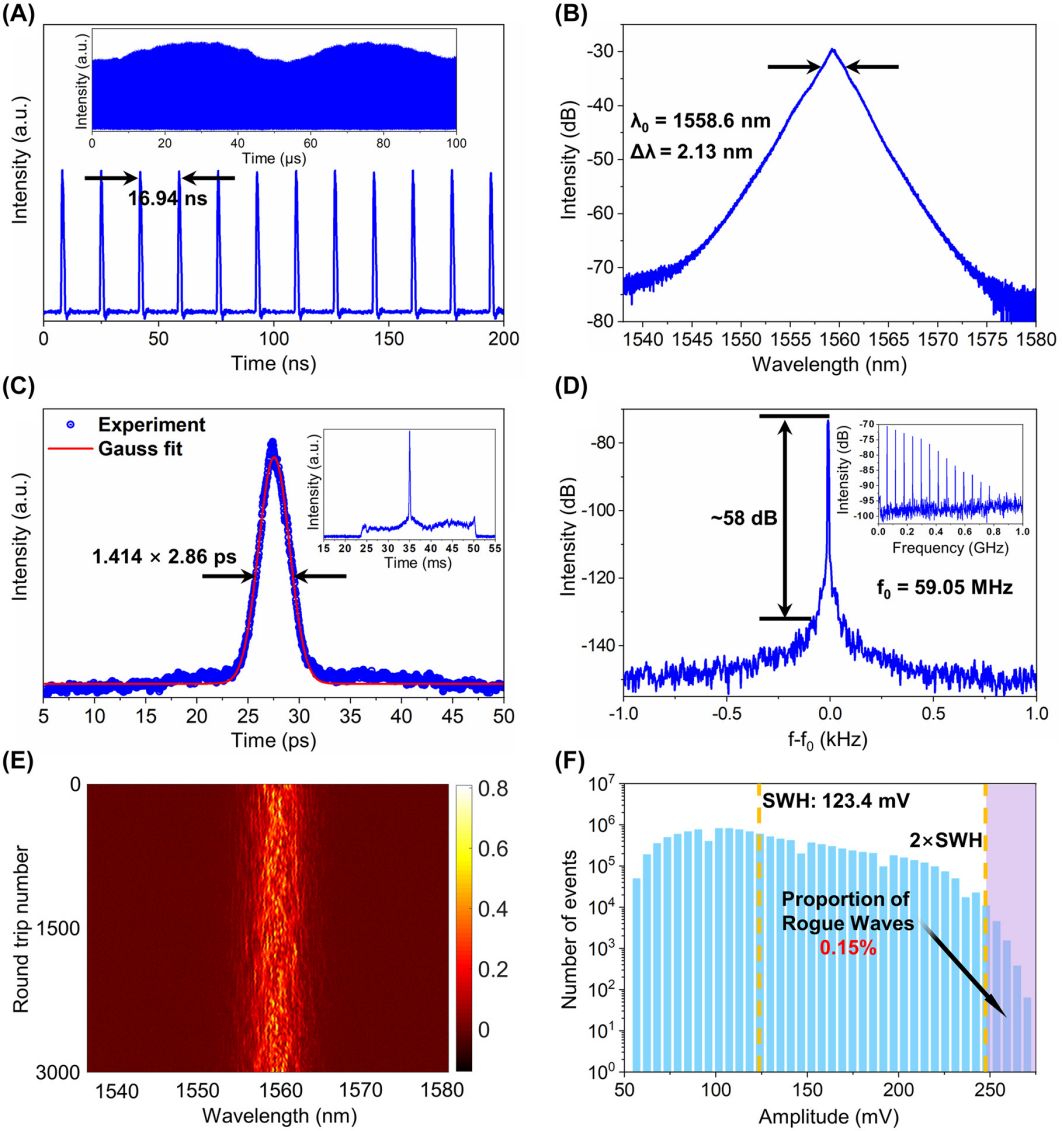
Noise-like mode-locked EDFL. (A) Oscilloscope trace; (inset: 100 μs pulse trains); (B) optical spectrum; (C) autocorrelation trace; (D) RF spectrum with 1 Hz resolution in the range of 2 kHz (inset: RF spectrum with 100 kHz resolution in the range of 1 GHz); (E) spectral evolution; (F) intensity histograms of noise-like pulses.

The total cavity length of the EDFL used in this experiment is 3.5 m, and the GVD in the cavity is calculated to be −0.063 ps^2^. Continuous-wave is obtained in the EDFL when the pump power is above 110 mW. When the pump power further reaches 180 mW, mode-locking could self-start, and then conventional soliton with fundamental repetition rate of 59.05 MHz is achieved. As the pump power increases, the stable soliton splits into several pulses. The fast intraband relaxation time by carrier–carrier introduces the attraction between multiple soliton pulses, which are impossible to be separated in time domain. Finally, pulses with random intensity and pulse width coexist with each other and gather tightly with smaller intervals to form noise-like pulses [37–39]. When the pump power is increased to 330 mW, EDFL is in a stable noise-like mode-locking state, and its output characteristics are shown in Figure 7. The average output power is 25 mW. As shown in Figure 7A, the interval between pulses is 24.82 ns, corresponding to a repetition rate of 59.05 MHz, consistent with the total cavity length, indicating that the mode-locked laser is operating at the fundamental frequency. The average single pulse energy is calculated to be 0.42 nJ, higher than that of conventional solitons. Obvious fluctuations of the pulse amplitude can be observed from the inset of Figure 7A. A pyramid-shaped smooth spectrum with a central wavelength of 1558.6 nm and a 3 dB bandwidth of 2.13 nm is observed, characteristic of noise-like operation. And it is shown in the autocorrelation trace in Figure 7C that there is a coherent peak of 2.86 ps on a broad base, which is typical for noise-like pulses. The TBP calculated from the spectral bandwidth and pulse duration is 0.75, slightly larger than the transform limit for Gaussian pulses, indicating that the obtained pulses are slightly chirped. The SNR reaches 58 dB, demonstrating that the mode-locked laser operates stably. The real-time spectral evolution of the noise-like pulse is shown in Figure 7E, showing fluctuations between the adjacent roundtrips. In order to estimate whether rogue waves are generated, about 7.37 million pulses are continuously recorded using a high-speed oscilloscope, and the energy fluctuation of the pulses is quantitatively analyzed. The histogram of pulse energy is shown in Figure 7F. Compared with the Gaussian distribution, the energy distribution shifts significantly to the high amplitude part. After statistical calculation, significant wave height (SWH), defined as the mean amplitude of the highest third of all the waves, is measured to be 123.4 mV. The highest recorded amplitude is 270.3 mV, nearly 2.2 times that of SWH [40, 41]. A wave with a peak power greater than twice that of SWH is usually referred to as a rogue wave, so it can be concluded that rogue waves are formed in this operation of noise-like pulses. And the proportion of rogue waves is statistically calculated to be 0.15%.

#### 5.2.2 7th harmonic mode-locking in the mode-locked EDFL

**Figure 8:**
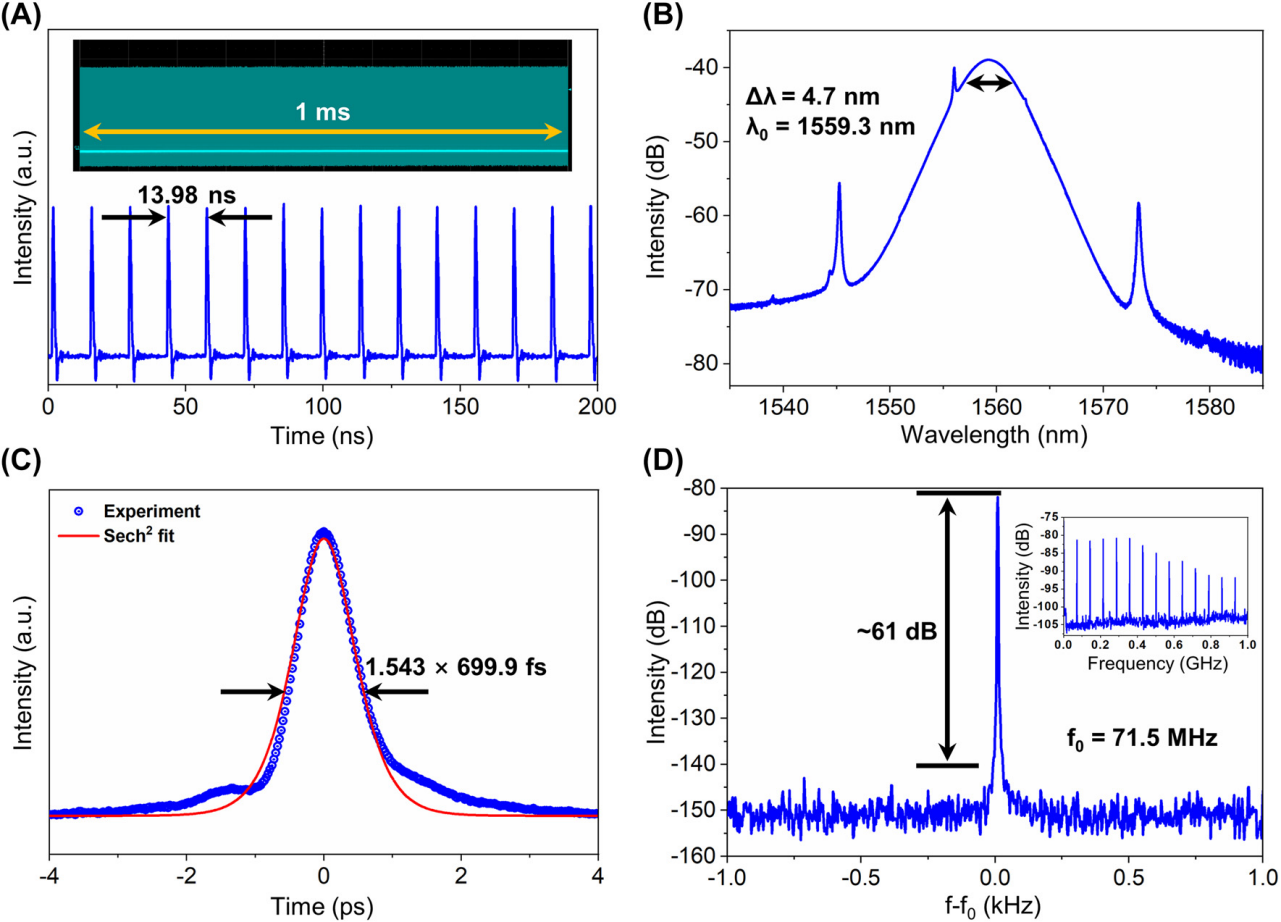
7th harmonic mode-locked EDFL. (A) Oscilloscope trace; (inset: 1.0 ms pulse trains); (B) optical spectrum; (C) autocorrelation trace; and (D) RF spectrum with 1 Hz resolution in the range of 2 kHz (inset: RF spectrum with 100 kHz resolution in the range of 1 GHz).

The cavity length of EDFL is increased to 20.22 m, with the gain fiber and germanene-SA sample remaining unchanged, and the GVD is up to −0.446* *ps^2^. The increase of pump power will cause excessive nonlinear phase shift in the resonant cavity with large anomalous dispersion, resulting in pulse splitting. Subsequently, the split pulses are separated by the repulsive force between adjacent pulses dominated by dispersive wave, gain depletion & recovery and acoustic-wave. Using the optoacoustic effect that induces a trapping potential, the acoustic resonance can stabilize the mode-locked state at different harmonics operation in the MLFL [42]. In this experiment, when the pump power is set to be 440 mW, harmonic mode-locking is obtained and its average output power is 15 mW. Figure 8A shows the mode-locked pulse trains recorded by the oscilloscope. The pulse interval is 13.98 ns, which is 1/7 of the pulse interval corresponding to the cavity length. Therefore, it can be concluded that the 7th order harmonic mode-locking pulses are obtained. The harmonic mode-locking pulse trains in the range of 1.0 ms are shown in the inset of Figure 8A, from which it can be seen that the pulse amplitude does not change significantly, meaning that the harmonic mode-locking laser operates stably. Figure 8B is the output spectrum, where the tilt is caused by the asymmetry of gain and loss, with central wavelength of 1559.3 nm and 3 dB bandwidth of 4.7 nm. It is obvious that the Kelly sidebands are symmetrically distributed in the spectrum. Due to the soliton area theorem, the energy of the pulse is limited, and the excess energy in the cavity exists in the form of dispersive wave. The dispersive wave interferes with the soliton, resulting in Kelly sidebands in the spectrum [43]. The autocorrelation trace of the harmonic mode-locking pulses is shown in Figure 8C. A good fit is obtained using the sech^2^ pulse profile, showing a pulse duration of 699.9 fs. The TBP is calculated to be 0.406, proving that the pulses are slightly chirped. As depicted from Figure 8D that the repetition rate of the mode-locked pulses is 71.5 MHz and the SNR reaches 61 dB. The peaks of integer multiples of the repetition rate in the 1 GHz range, shown in the inset, remain uniform and stable. This demonstrates the stability of the harmonic mode-locking.

When the pump power reaches the maximum output power of 440 mW, the above two mode-locked EDFL could operate normally without damage of the SA.

### 5.3 Mode-locked TDFL characterization

**Figure 9:**
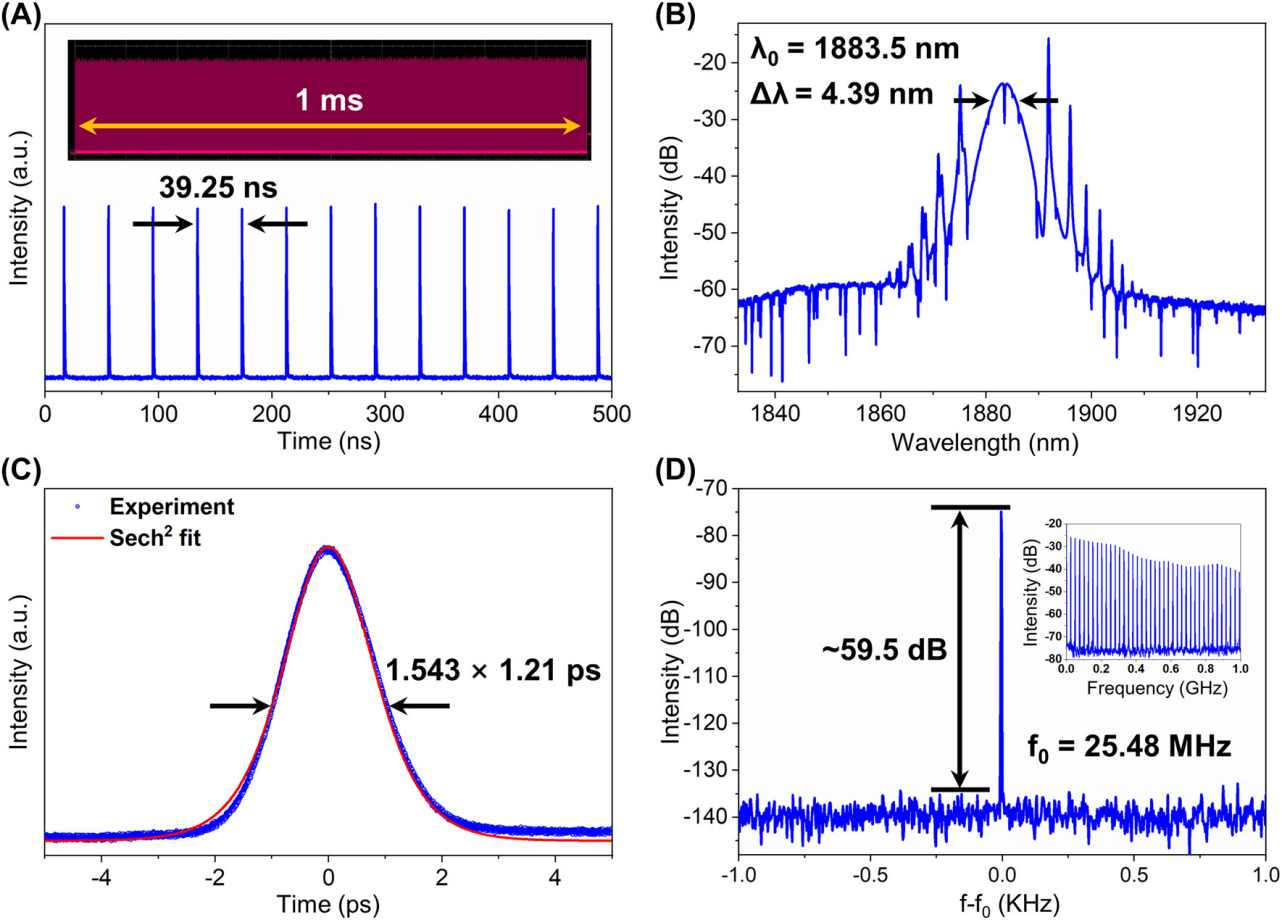
Conventional solitons for mode-locked TDFL. (A) Oscilloscope trace; (inset: 1.0 ms pulse trains); (B) optical spectrum; (C) autocorrelation trace; and (D) RF spectrum with 1 Hz resolution in the range of 2 kHz (inset: RF spectrum with 100 kHz resolution in the range of 1 GHz).

In order to verify the broadband saturation absorption property of germanene-SA, a TDFL is constructed. The total cavity length of the TDFL is 8.14 m, and its GVD is calculated to be −0.603 ps^2^. The performance of the mode-locked TDFL at a pump power of 972 mW, with the average output power of 18 mW, is presented in Figure 9. As can be seen from Figure 9A, the time interval between pulses is 39.25 ns, consistent with the cavity length, indicating the fundamental repetition rate operation. The high stability of the mode-locked pulses is confirmed from the 1.0 ms pulse trains in the inset with no obvious fluctuation. The spectrum of the soliton pulses is shown in Figure 9B, where the central wavelength and 3 dB bandwidth are 1883.5 and 4.39 nm, respectively. Figure 9C shows the shape of the output pulses, and the pulse duration is fitted as 1.21 ps. The TBP is calculated to be 0.449, which is close to the theoretical value (0.315) for the transform-limited sech^2^-shaped pulses. The SNR of the RF spectrum, with 1 Hz resolution shown in Figure 9D, is 59.5 dB at the fundamental repetition rate of 25.48 MHz, indicating stable mode-locking. In the inset of Figure 9D, the range of measurement is set to be 1 GHz with a resolution bandwidth of 100 kHz. When the pump power is gradually increased to 1.43 W, the emission peak in the optical spectrum of TDFL disappears, leaving only a wide smooth substrate. And it is indicated that the damage threshold of germanene-SA has been reached, resulting in the failure of TDFL operation.

We also found that after the germanene-SA is stored in the air for about one week, its saturable absorption characteristics will disappear, resulting in the inability to achieve ultrashort pulses.

## 6 Conclusions

In summary, germanium nanosheets are fabricated by LPE method, and are deposited on the facet of an optical fiber connector using laser-induced deposition method to assemble the germanene-SA. The saturable absorption of germanene-SA is characterized by balanced synchronous twin-detector measurement, and the MDs at 1.0, 1.5 and 2.0 μm bands are calculated to be 4.19, 6.75, and 8.45%, respectively. By inserting germanene-SA into the YDFL, EDFL and TDFL, the mode-locked pulses in three major bands in the range of 1.0–2.0 μm are achieved. Dissipative soliton pulses and conventional soliton pulses are obtained at 1061.1 and 1883.5 nm with pulse durations of 79.6 and 1.21 ps, respectively. Two operating states are realized at 1.5 μm band including noise-like mode-locking and conventional soliton mode-locking. In addition, the existence of rogue waves is further observed in the operation of noise-like pulses, and the proportion of rogue waves is 0.15% by statistical calculation. To the best of our knowledge, it is the first demonstration of the broadband (up to 1000 nm) operation property of the germanene-SA, laying an important foundation for the application in the broadband ultrafast lasers. In addition, germanene-SA is also expected to realize mid-infrared MLFLs, which have important applications in the environmental monitoring, atmospheric optical communication and special materials processing.

## Supplementary Material

Supplementary Material Details

Supplementary Material Details
