# Supplementary material for: Broadband saturable absorption in germanene for mode-locked Yb, Er, and Tm fiber lasers
Source: Nanophotonics. 2022 May 17;11(13):3127–37. doi: 10.1515/nanoph-2022-0161 (PMC11501555; doi:10.1515/nanoph-2022-0161)
Supplement: Supplementary file 1 — Supplementary Material Details [file j_nanoph-2022-0161_suppl_001.docx]

Pan Wang

Institute of Modern Optics

Nankai University

300350 Tianjin, China

pan.wang@nankai.edu.cn

Tianjin, 3/18/22

Dear Editor,

Please consider our manuscript entitled “Broadband saturable absorption in germanene for mode-locked Yb, Er, and Tm fiber lasers” for publication in ***Nanophotonics***.

Ultrafast fiber lasers have been proved as the most effective tool for various crucial fields, such as strong-field physics, nonlinear optics, precision metrology, ultrafine machining, and industrial processing. Among them, passively mode-locked fiber lasers (MLFLs) stand out in the past decades, with the advantages of compact structure, self-starting operation, high stability, and free maintenance. Importantly, saturable absorber (SA) is a necessary part in passively MLFLs, which can be divided into artificial SA and real SA. Fiber laser systems built with real SA tend to have simpler structures and lower costs. Meanwhile, the research work about real SA is in the frontier and interdisciplinary fields of many disciplines such as physics, chemistry and material science. Therefore, the exploration of real SA has been a hotspot in the research of MLFLs in recent years.

Since Bao et al. used graphene as real SA to achieve ultrashort pulses at 1565 nm in 2009, it has become the focus of scientists to explore novel suitable real SA based on 2D materials, which have ultrafast carrier dynamics, 2D planar structures and broadband response. Although a group of 2D materials have been successfully used as broadband SAs for MLFLs, such as graphene, black phosphorus, transition metal dichalcogenides, topological insulators, and MXenes, there are still some deficiencies that limit their practical applications. Very recently, germanene has been shown to be a graphene-like Dirac material with ultrafast optical response and broadband optical absorption property, revealing promising applications in optoelectronic and photonic applications. Since the germanene layer is a natural quantum well, and the semiconductor layer acts as a reservoir to provide a relevant charge flow to the germanene layer, the carriers in the layer have a much shorter relaxation time. In addition, germanene also has a much higher nonlinear absorption coefficient than graphene. Therefore, germanene has great potential as a fast-SA for ultrafast photonics.

In this paper, germanium nanosheets are prepared by liquid-phase exfoliated method, and then the laser-induced deposition method is used to assemble the germanene-SA. Next, we systematically investigate the optical property of the SA and verify its saturable absorption operation in all major fiber lasers (i.e., Yb-, Er-, and Tm-doped fiber lasers) in the wavelength range of 1.0~2.0 μm. Stable ultrashort pulses are realized at 1061.1 nm, 1559.3 nm and 1883.5 nm, respectively. To the best of our knowledge, this is the first demonstration of the broadband (up to ~1000 nm) operation property of germanene-SA, capable of generating ultrashort pulses covering three key wavelengths (i.e., 1.0, 1.5 and 2.0 μm).

For these reasons we feel the paper fulfils the editorial guidelines of ***Nanophotonics*** and will be of great interest to the broad community of ultrafast photonics, fiber optics, Nonlinear fiber optics and nanophotonic devices.

We have 8 authors in this paper. The contacts of each author are listed below:

1. Qingbo Wang, Institute of Modern Optics, Nankai University, Tianjin Key Laboratory of Micro-scale Optical Information Science and Technology, Tianjin 300350, China

E-mail: qbwang578@163.com

2. Jianlong kang, College of Physics and Optoelectronic Engineering, Shenzhen University, Shenzhen 518060, China

E-mail: kangjianlong2018@email.szu.edu.cn

3. Pan Wang, Institute of Modern Optics, Nankai University, Tianjin Key Laboratory of Micro-scale Optical Information Science and Technology, Tianjin 300350, China

E-mail: pan.wang@nankai.edu.cn

4. Jiangyong He, Institute of Modern Optics, Nankai University, Tianjin Key Laboratory of Micro-scale Optical Information Science and Technology, Tianjin 300350, China

E-mail: 1120190097@mail.nankai.edu.cn

5. Yicong Liu, Institute of Modern Optics, Nankai University, Tianjin Key Laboratory of Micro-scale Optical Information Science and Technology, Tianjin 300350, China

E-mail: 2013472@mail.nankai.edu.cn

6. Zhi Wang, Institute of Modern Optics, Nankai University, Tianjin Key Laboratory of Micro-scale Optical Information Science and Technology, Tianjin 300350, China

E-mail: zhiwang@nankai.edu.cn

7. Han Zhang, College of Physics and Optoelectronic Engineering, Shenzhen University, Shenzhen 518060, China

E-mail: hzhang@szu.edu.cn

8. Yange Liu, Institute of Modern Optics, Nankai University, Tianjin Key Laboratory of Micro-scale Optical Information Science and Technology, Tianjin 300350, China

E-mail: ygliu@nankai.edu.cn

Suggested reviewers:

- Philippe Grelu, Director, Laboratoire ICB UMR 6303 CNRS, Université Bourgogne Franche-Comté, Dijon, France [philippe.grelu@u-bourgogne.fr]
- Xiaosheng Xiao, State Key Laboratory of Information Photonics and Optical Communications, School of Electronic Engineering, Beijing University of Posts and Telecommunications, Beijing, 100876, China [xsxiao@bupt.edu.cn]
- Aiping Luo, Professor, Guangdong Provincial Key Laboratory of Nanophotonic Functional Materials and Devices & Guangzhou Key Laboratory for Special Fiber Photonic Devices and Applications, South China Normal University, Guangzhou, 510631, China [luoaiping@scnu.edu.cn]
- Shiming Gao, Professor, Centre for Optical and Electromagnetic Research, State Key Laboratory for Modern Optical Instrumentation, Zhejiang University, Zhejiang, Hangzhou, 310058, China [gaosm@zju.edu.cn]
- Chengbo Mou, Professor, Key Lab of Specialty Fiber Optics and Optical Access Network, Joint International Research Laboratory of Specialty Fiber Optics and Advanced Communication, Shanghai Institute for Advanced Communication and Data Science, Shanghai University, Shanghai, 200072, China [mouc1@shu.edu.cn]

Yours sincerely,

Pan Wang
